# Sociocultural Attitudes Toward Appearance and Attitudes Toward Eating in Adolescents With Type 1 Diabetes: The Importance of Perfectionism

**DOI:** 10.1155/pedi/9993342

**Published:** 2025-01-31

**Authors:** Desireé Ruiz-Aranda, Ana Luque, Francesca Russo, Javier Fenollar-Cortés

**Affiliations:** ^1^Department of Psychology, Loyola University Andalusia, Seville, Spain; ^2^Department of Psychology, European University of Rome, Rome, Italy; ^3^Department of Psychology, European University of Valencia, Valencia, Spain

**Keywords:** adolescents, eating disorders, perfectionism, type 1 diabetes

## Abstract

**Objective:** Adolescents managing type 1 diabetes (T1D) are at increased risk of experiencing eating disorders (EDs). Identifying risk factors is essential to develop preventive strategies. This study examines the potential mediation value of self-esteem and the perfectionism associated with EDs in the relationship between sociocultural attitudes toward appearance and eating attitudes related to EDs in a sample of adolescents with T1D.

**Methods:** Forty-six adolescents aged 12–17 years diagnosed with T1D participated in the current study. Sociocultural attitudes toward appearance, perfectionism associated with EDs, and self-esteem were measured. Multiple and simple mediator analyses using the bootstrapping method with bias-corrected confidence estimates were conducted.

**Results:** Our results show that perfectionism associated with eating problems is not only related to sociocultural attitudes toward appearance and eating attitudes, but rather the relationship between these last two variables would be fully mediated by perfectionism.

**Conclusions:** A high degree of perfectionism could be a risk variable when developing potential eating problems in T1D adolescents. Perfectionism and its self-management would be a prominent factor that may help to design interventions developed for adolescents with diabetes who show behaviors that potentially conflict with eating. The clinical implications are discussed.

## 1. Introduction

Type 1 diabetes (T1D) is a chronic and autoimmune disorder associated with β-cell dysfunction, characterized by an abnormal production of insulin, resulting in high blood glucose values [1]. Generally, the onset of T1D occurs in childhood or adolescence. However, recent studies showed it could also develop during adulthood [2]. The global incidence of this disorder among adolescents is dramatically increasing, with estimates of 108.300 children under 15 years newly diagnosed with T1D in 2021 [3].

Managing T1D in children and adolescents necessitates the adoption of specific daily management behaviors. This includes the structured administration of insulin, routine blood glucose monitoring, and adherence to a prescribed eating plan. Additionally, regular sleep patterns and physical activity are integral to maintaining glycemic outcome and overall health [4]. These adjustments, often required for medical reasons, go beyond voluntary lifestyle choices and are crucial for effective disease management. Frequently, significant weight loss immediately occurs before diagnosis, followed by rapid weight regain with the initiation of insulin treatment, which may increase concerns about weight and body shape in adolescents. For these reasons, people living with T1D are at increased risk of experiencing eating disorders (EDs), particularly during childhood or adolescence [5]. Moreover, the management of insulin therapy and carbohydrate control may be additional trigger factors [6]. EDs in individuals with T1D occur at approximately twice the rate observed in the general population [7]. The prevalence of EDs in diabetic adolescents is about 7%, with severe consequences both in terms of higher glycated hemoglobin values and acute and chronic complications [8].

In addition to these specific risk factors, individual variables and sociocultural factors related to the idealization of thinness have traditionally been most associated with EDs [9]. On the one hand, perfectionism is understood as a multidimensional personality trait characterized by pursuing perfection, setting excessively high-performance standards, and extreme self-critical evaluation [10]. In the general population, perfectionism has been identified as a risk factor for developing EDs [11, 12]. It is also considered a predictor of adverse outcomes in treating these disorders [13]. For people with T1D, pressure from medical teams and family to achieve sometimes unattainable glucose outcomes, avoidance of certain foods, and increased attention to weight changes were factors that contributed to the development of rigid standards around eating and weight that can exacerbate EDs [14].

On the other hand, self-esteem is related to the onset and maintenance of EDs. Some studies suggest that low self-esteem could be a transdiagnostic predictor for EDs. [15], considering that low self-esteem and eating pathology would reciprocally affect each other. The symptoms of EDs could lead to enduring damage in the individual's self-concept in a vicious cycle of maintenance [16].

Appearance-related pressures and thin-ideal internalization are well-established risk factors for body dissatisfaction and disordered eating among adolescents [17]. The sociocultural theory is a valuable framework for exploring how environmental influences contribute to body image concerns [18]. Research findings consistently support the strong relationship between sociocultural influences and body dissatisfaction [19]. Perfectionistic traits may increase the tendency to perceive pressures to be thin and susceptibility to internalize appearance ideals, which could subsequently increase vulnerability to ED symptoms [20].

Although most studies have explored these factors separately [21, 22], few studies have examined how personality variables, like perfectionism and self-esteem, work in combination with sociocultural factors to predict individual differences in EDs [20]. The current study aims to assess a model of risk for engaging in EDs in adolescents with T1D to identify risk for EDs and the mechanisms by which perfectionism and self-esteem might be associated with EDs in adolescents with T1D. Specifically, this study examines the potential mediation value of self-esteem and the perfectionism associated with EDs in the relationship between sociocultural attitudes toward appearance and eating attitudes related to EDs in a sample of adolescents with T1D.

## 2. Method

### 2.1. Participants and Procedure

The participants were 24 males (52.2%), 21 females (45.7%), and 1 nonbinary-defined person (2.2%) from 12 to 17 years (*M* = 15.0, standard deviation [SD] = 1.59) diagnosed with T1D. The mean and SD for the age of diagnosis, sports activities, HbA1c level, and all outcome measures are included in Table 1. From the entire sample, four participants were diagnosed with lactose intolerance, three participants were diagnosed with hypothyroidism, and two were diagnosed with celiac disease.

The sample was recruited from hospitals and families of adolescents with diabetes associations localized in Andalusia (Spain). The inclusion criteria were (a) to be diagnosed with T1D; (b) ages between 12 and 17; and (c) no concomitant ED diagnosis at the moment of the study. The participants' families were informed about the study and asked to collaborate by permitting minors to complete the measures. All the families signed the consent form once informed. Data are available on request. The study was approved by the Ethical Committee of the University of Loyola.

## 3. Measures

### 3.1. Background Characteristics

The following background characteristics were analyzed: age, age of diagnosis, sports activities (measured by the total hours by week dedicated to sports activities), HbA1c level, and concomitant medical illness. These background characteristics were selected by referring to the literature.

### 3.2. Sociocultural Attitudes Toward Appearance

The Spanish Version of the Sociocultural Attitudes Towards Appearance Questionnaire-4 (SATAQ-4 original version [23]; Spanish version [24]) was used to measure the degree of sociocultural influences on a person's body image. The SATAQ-4 is a 22-item questionnaire with Likert response items ranging from 1 (“completely disagree”) to 5 (“completely agree”). The scale measures five factors of sociocultural influences: three pressure subscales (i.e., parents, peers, and media), two internalization subscales (thin/low body fat and muscular/athletic), and a Total SATAQ-4 score. The analyses used only the Total SATAQ-4 score in the current study. The higher score indicates the more significant influence of social aspects on appearance. The Total SATAQ-4 score consistency was high (McDonald's *ω* = 0.95).

### 3.3. Eating Attitudes Related to ED

The eating attitudes were explored by the 26-item Eating Attitudes Test (EAT-26 [25]; Spanish version [26]). EAT-26 is a self-rated eating scale that measures symptoms and concerns of EDs. The EAT-23 includes three sections: (a) self-reported height and weight to calculate a body mass index, (b) 26 items rated on a 6-point Likert scale related to how often an individual engages in particular, and (c) five behavioral items on a 6-point Likert scale examining how often a person has engaged in disordered eating behaviors over the past 6 months. The current study only examined the 26 items of the EAT-26 and did not consider the other two sections. The items are rescored on a 6-point scale, corresponding to “always” (3 points), “usually” (2 points), “often” (1 point), “sometimes” (0 points), “rarely” (0 points), and “never” (0 points). The higher score indicates a higher ED risk. The consistency was good (McDonald's *ω* = 0.78).

### 3.4. Perfectionism Associated With EDs

The subscale “perfectionism” of the Eating Disorder Inventory (EDI [27]; Spanish version [28]) was used to measure perfectionism related to EDs. The original scale includes eight subscales: (1) drive for thinness, (2) bulimia, (3) body dissatisfaction, (4) ineffectiveness, (5) perfectionism, (6) interpersonal distrust, (7) interoceptive awareness, and (8) maturity fears. The items are rated as follows: “never” (0 points), “rarely” (0), “sometimes” (0), “often” (1 point), “usually” (2 points), and “always” (3 points). The current study only examined the 6-item perfectionism subscale. The higher score indicates a higher perfectionism level. The consistency was good (McDonald's *ω* = 0.80).

### 3.5. Self-Esteem

The Rosenberg Self-Esteem Scale (RSES [29]; Spanish version [30]) was used to measure self-esteem. The RSES is a 10-item scale widely used measure of global self-esteem and general feelings of self-worth. All items are answered using a 4-point Likert scale format ranging from 1 (“strongly agree”) to 4 (strongly disagree”). A higher score indicates a better self-esteem. The consistency was good (McDonald's *ω* = 0.80).

### 3.6. Data Analyses

The data distribution was explored by inspecting visual Q–Q plots and calculating the *Z*-statistic for skewness and kurtosis values. Given the sample size, *Z*-values higher than |1.96| were considered indicators of non-normal distribution [31]. Independent *t*-tests and Mann–Whitney analyses were conducted to explore if sex would be introduced into the models as a covariate. The single nonbinary-defined person was excluded from the sex comparison analysis to make interpreting the results more accessible. The reliability of the scales was measured by McDonald's omega statistics [32]. Internal consistency is usually considered acceptable if McDonald's *ω* is 0.70 or higher [33].

Zero-order intercorrelations between all outcome measures were computed to substantiate the consideration of possible indirect influences of perfectionism and self-esteem in the relationship between sociocultural attitudes toward appearance and eating attitudes. All significantly correlated variables were retained to be included in the mediation analyses. To check if the age, the age of diagnosis, the HbA1c levels, or the sports activities needed to be controlled for in later analyses, their correlations with the outcome measures were also examined. A correlation coefficient of 0.10 was considered a small effect, 0.30 was considered a medium effect, and 0.50 was considered a large effect [34].

To examine if perfectionism and self-esteem mediated the effects of sociocultural attitudes toward appearance on eating attitudes, we performed serial multiple mediator models using the bootstrapping method with bias-corrected confidence estimates [35], which minimize type 2 error [36]. Bootstrapping was used to establish the statistical significance of all totals, direct, and indirect effects. The effect is considered significant if the upper and lower bounds of the bias-corrected 95% confidence intervals do not contain zero. This method does not impose the assumption of normality of sampling distribution. We used 5000 bootstrap samples for both simple and multiple mediator models. The mediation analyses were conducted using the PROCESS macro for IBM SPSS [37].

A serial multiple mediation model was conducted to explore if the perfectionism associated with eating problems and self-esteem mediated the effects of sociocultural attitudes toward appearance on eating attitudes. Finally, simple mediation models were also tested to explore whether the direct and indirect effects are still significant even without the presence of the other mediators.

## 4. Results

Data distribution analyses showed that the following variables did not meet the normality assumptions: sports activities (*Z*_Kurtosis_ = 5.90), eating attitudes (*Z*_Skewness_ = 5.90), and perfectionism (*Z*_Skewness_ = 4.10). There were no significant differences between sex either for the age (*t* (43) = −0.09, *p*=0.927), the age of diagnosis (*t* (43) = −1.71, *p*=0.095), or sports activities (*U* = 281, *p*=0.511), or HbA1c level (*t* (43) = 0.32, *p*=0.754). Regarding the outcome variables, no significant differences were found for eating attitudes (*U* = 215, *p*=0.404), sociocultural attitudes toward appearance (*t* (43) = −1.27, *p*=0.210), or perfectionism (*U* = 198, *p*=0.222).

The results of the correlation analysis and the descriptive statistics are summarized in Table 1, which displays the relationships between the key variables discussed in the Results section. As expected, the eating attitude variable was correlated with sociocultural attitudes toward appearance (ρ = 0.34, *p*=0.021) and perfectionism (ρ = 0.42, *p*=0.003). The sociocultural attitudes toward appearance were correlated with self-esteem (*r* = −0.44, *p*=0.002) and perfectionism (ρ = 0.41, *p*=0.005). Given that the age correlated with perfectionism (ρ = 0.54, *p* < 0.001) and the age of diagnosis correlated with self-esteem (*r* = −0.34, *p*=0.020), both variables were entered as covariates in the mediation analyses. No significant relationship has been found between HbA1C level and perfectionism, eating attitudes, self-esteem, or sociocultural attitudes toward appearance.

### 4.1. Mediating Roles of Perfectionism and Self-Esteem in the Relationship Between Sociocultural Attitudes Toward Appearance on Eating Attitudes

**Figure 1:**
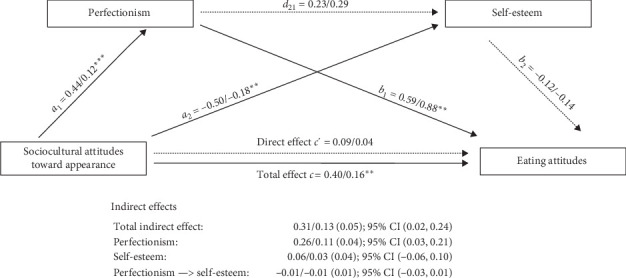
Serial mediation model linking sociocultural attitudes toward appearance (SATAQ total score) and eating attitudes (EAT), in which perfectionism (EDI) and self-esteem (RSES) are the mediator variables. *Note:* The dashed arrows are nonsignificant. Both the age and the age of diagnosis variables were included in the model as covariates. Standardized and unstandardized regression coefficients are presented sequentially for each direct effect (i.e., *β*/*B*). The bootstrapped standard errors are presented in the parenthesis. *⁣*^*∗*^*p* < 0.05; *⁣*^*∗∗*^*p* < 0.01; *⁣*^*∗∗∗*^*p* < 0.001.

Regression coefficient estimates and bias-corrected 95% confidence intervals for the indirect effects of perfectionism and self-esteem in the relationship between sociocultural attitudes toward appearance and eating attitudes are presented in Figure 1. Results indicated that whereas perfectionism had a unique effect on eating attitudes (*B* = 0.88, *t* (46) = 3.26, *p*=0.002), self-esteem did not (*B* = −0.14, *t* (46) = −0.80, *p*=0.432). Results of the multiple mediation analyses confirmed that the relationship between sociocultural attitudes toward appearance and eating attitudes would be mediated by perfectionism, conditional on the presence of self-esteem in the model, as well as the age and age of diagnosis as covariates (indirect effect = 0.13 [0.05], 95% CI [0.02, 0.24]). That is, sociocultural attitudes toward appearance were positively related to perfectionism (*β* = 0.44, *p* < 0.001), which was positively related to eating attitudes (*β* = 0.59, *p*=0.002).

However, the indirect effects provided for the sequential mediation model suggest that only one pathway would be relevant. Except for perfectionism as a single mediator variable between the relationship between sociocultural attitudes toward appearance and eating attitudes (indirect effect = 0.11 [0.04], 95% CI [0.03, 0.21]), the rest of the indirect effects were not significant (Figure 1).

**Figure 2:**
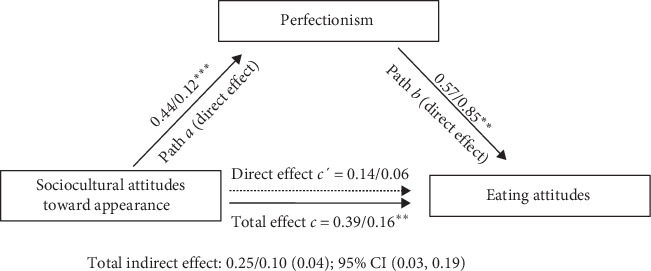
Simple mediation model linking the sociocultural attitudes toward appearance (SATAQ total score) and eating attitudes (EAT), in which perfectionism is the mediator variable. *Note:* The dashed arrows are nonsignificant. The age was included as a covariate in the model. Standardized and unstandardized regression coefficients are presented sequentially for each direct effect (i.e., *β*/*B*). The bootstrapped standard errors are presented in the parenthesis. *⁣*^*∗*^*p* < 0.05; *⁣*^*∗∗*^*p* < 0.01; *⁣*^*∗∗∗*^*p* < 0.001.

The results of the simple mediation models were in line with the multiple mediation model results. The indirect effect of sociocultural attitudes toward appearance on eating attitudes through perfectionism (the age as a covariate) was still significant (indirect effect = 0.10 [0.04], 95% CI [0.03, 0.19]) (Figure 2). That is, the relationship between sociocultural attitudes toward appearance and eating attitudes was fully mediated by perfectionism. Sociocultural attitudes toward appearance were positively related to perfectionism (*β* = 0.44, *p* < 0.001), which was positively related to eating attitudes (*β* = 0.57, *p*=0.002). The result of the simple mediation model to explore the indirect effects of sociocultural attitudes toward appearance on eating attitudes through self-esteem (the age of diagnosis as a covariate) was conducted. As expected, the indirect effect was no significant (indirect effect = 0.00 [0.03], 95% CI [−0.08, 0.06]).

## 5. Discussion

The main goal of the current study was to explore the potential mediation value of self-esteem and the perfectionism associated with EDs in the relationship between sociocultural attitudes toward appearance and eating attitudes related to EDs in a sample of adolescents with T1D. Determining possible mediating variables could help us to identify relevant variables for the early detection of EDs, as well as to explore mechanisms by which perfectionism might be associated with EDs in adolescents with T1D. Our results show that perfectionism associated with eating problems would not only be related to both sociocultural attitudes toward appearance and EDs but rather the relationship between these last two variables would be entirely mediated by perfectionism. No significant relationships were found between metabolic control and EDs. These results are consistent with results of studies longitudinal studies that indicate that patients with EDs do not see reflected alterations in their metabolic control until they are at least several years of evolution [38].

Although previous studies point out the relationship between attitudes toward appearance and attitudes toward eating [17, 20], our findings seem to show that this association has a specific nature in the group of adolescents with diabetes and provide preliminary support for an intriguing hypothesis about how perfectionism, which might lead to the development of ED symptoms. Perfectionism may increase vulnerability to sociocultural messages related to thinness and, therefore, would increase the risk of developing specific eating problems. High levels of perfectionism are a critical feature in some transdiagnostic models of EDs [39], and this trait may increase the risk for the development of EDs in individuals with T1D [40]. In these adolescents, perfectionism may manifest as a tendency to set unrealistic goals regarding disease management, creating unattainable expectations in both glucose targets and body weight. As a result, the inability to meet these goals fosters a sense of failure, reinforcing body dissatisfaction and the perception of inefficacy, both factors strongly associated with EDs [14]. Moreover, the repeated review of diabetes management, along with the fear of not meeting the set goals—whether established by the medical team or self-imposed—can gradually compromise the mental health of these adolescents, exacerbating disordered eating behaviors. This connection between perfectionism, self-imposed demands in diabetes management, and the development of EDs highlights the importance of addressing perfectionism in preventive and therapeutic interventions for this population. The pattern of preoccupation with eating and meal planning necessary for diabetes management (aspects of the treatment regimen that require a greater focus on food and eating), along with the risk of increased body weight, negative feelings about shape, and body weight, can become a complex challenge for adolescents with diabetes [41]. Psychosocial assessments should be integrated into routine diabetes care, including screening for symptoms of disordered eating in adolescents with diabetes [42]. This should be done at the initial visit, at periodic intervals, and when there are changes in disease management, treatment, or life circumstances. Early detection and intervention are crucial to improving the prognosis of EDs [43]. In clinical practice, healthcare providers must monitor several potential indicators of eating difficulties, including changes in eating habits, mood disturbances, weight fluctuations, self-esteem, and body image distortions. Our study highlights the importance of considering perfectionism as a significant risk factor in the development of EDs in adolescents with T1D.

On the contrary, although self-esteem was related to sociocultural attitudes toward appearance, the model did not allow us to conclude that it had a mediation value between this last variable and attitudes toward eating. Similarly, the diabetes eating problem variable was related to sociocultural attitudes toward appearance but did not show any mediation value.

Several limitations of the study must be acknowledged. First, it has not been taken into account the distinction between two broad dimensions of perfectionism that include adaptive and maladaptive components, respectively, referred to as perfectionistic strivings and perfectionistic concerns [44]. The multidimensional approach can detect specific facets of perfectionism and their relationship with the risk of EDs. Thus, using instruments that assess a general disposition may not capture the different effects of perfectionism dimensions on EDs [12]. Second, this study relied on self-report measures. Although the measures are well-validated, participant responses could be susceptible to biases. In future research, it would be essential to include clinical interviews and parent reports to reduce response biases.

The study has some clinical implications. EDs in young adolescents with illness have severe health problems that, in most cases, go unnoticed [8]. To avoid such a significant delay in diagnosis, and despite the limitations of the present study, clinicians must remain alert and closely screen patients with T1D for EDs. The pattern of preoccupation with food and meal planning necessary for diabetes management, along with the risk of higher body weight, negative feelings about body shape and weight, chronically elevated blood sugars, depression, anxiety, shame, and poor diabetes self-care is complex and challenging for the patient and the clinician alike [45]. Early detection of risk factors and access to early intervention are essential in improving and maintaining the quality of health and well-being for adolescents with diabetes.

### 5.1. Clinical Implications

That is why clinicians need to be aware of the early risk factors associated with changes in eating patterns and warning signs that EDs may have already become established in their patients. Moreover, in the case of adolescents with diabetes, a high degree of perfectionism could be a risk variable when developing potential eating problems, so in addition to considering this variable in the entire general adolescent population, perfectionism and its self-management would be the subject of special clinical attention in adolescents with diabetes who show behaviors that potentially conflict with eating. Prevention and intervention efforts to address perfectionism and the tendency to perceive pressures as thin and susceptibility to internalize appearance ideals are critical to improving the psychosocial and physical health of adolescents with T1D.

## Figures and Tables

**Table 1:** Descriptive statistics and intercorrelations among variables (*N* = 46).

Variables	1	2	3	4	5	6	7	8
1. Perfectionism^a^	—	—	—	—	—	—	—	—
2. Eating attitudes^a^	0.53^*∗∗∗*^	—	—	—	—	—	—	—
3. Self-esteem	−0.01	−0.16	—	—	—	—	—	—
4. Sociocultural attitudes toward appearance	0.51^*∗∗∗*^	0.40^*∗∗*^	−0.44^*∗∗*^	—	—	—	—	—
5. Age	0.54^*∗∗∗*^	0.10	−0.06	0.15	—	—	—	—
6. HbA1C level	−0.01	0.16	−0.21	0.06	0.23	—	—	—
7. Age of diagnosis	0.02	−0.05	−0.34^*∗*^	0.11	−0.02	−0.24	—	—
8. Sport^a^	0.06	−0.02	−0.06	0.10	−0.09^*∗∗∗*^	−0.15^*∗∗∗*^	0.03^*∗∗∗*^	—

Mean	7.22	6.65	31.4	46.7	15.0	7.58	9.15	3.44
SD	4.73	7.04	6.03	17.1	1.49	0.99	3.72	3.86

Abbreviation: SD, standard deviation.

^a^Correlations for these variables were calculated using Spearman's correlation coefficients instead of Pearson's due to the non-normal distribution of the data.

*⁣* ^*∗*^ *p* < 0.05; *⁣*^*∗∗*^*p* < 0.01; *⁣*^*∗∗∗*^*p* < 0.001.

## Data Availability

Data are available on request from the authors.
